# Factors Influencing the Preference of Medical Students at Umm Al-Qura University for Otorhinolaryngology-Head and Neck Surgery as a Future Specialty: A Cross-Sectional Study

**DOI:** 10.7759/cureus.58917

**Published:** 2024-04-24

**Authors:** Saad M Alharthi, Bader Al-Kaabi, Shaimaa K Alnajjar, Raghad Y Shosho, Ameera A Alkhamesi, Abdulrahman F Kabli, Ahmed Alzahrani, Lina F Serhan, Mokhtar Shatla

**Affiliations:** 1 General Practice, King Faisal Specialist Hospital, Makkah, SAU; 2 Medicine and Surgery, Umm Al-Qura University, Makkah, SAU; 3 Medicine, Umm Al-Qura University, Makkah, SAU; 4 Medicine, King Abdulaziz University, Jeddah, SAU; 5 Otolaryngology-Head and Neck Surgery, King Abdullah Medical City, Makkah, SAU; 6 Medicine, Almaarefa University, Riyadh, SAU; 7 Family Medicine, Umm Al-Qura University, Makkah, SAU

**Keywords:** work hours, hospital, choice of career, surgery, specialization

## Abstract

Background: Otorhinolaryngology (ORL) specialists treat patients of different ages, ranging from elderly patients with head and neck tumors to neonates with respiratory problems. No studies have been conducted to explore the factors that affect the preference for ORL among Umm Al-Qura University students. We aimed to investigate factors and motivators influencing medical students at Umm Al-Qura University in Makkah, Saudi Arabia, who choose to pursue a career in ORL-head and neck surgery.

Methods and materials: This cross-sectional study was conducted over two months in 2023 among 439 students in the pre-clinical, clinical, and internship years of the Faculty of Medicine at Umm Al-Qura University, Saudi Arabia. We shared a Google Forms questionnaire (Google, Inc., Mountain View, CA) and explored factors associated with interest in ear, nose, and throat (ENT) as a future specialty. We used Pearson's chi-square test to arrive at the results.

Results: A total of 339 (77.2%) participants were female. Participants were evenly divided between pre-clinical years (213 (48.5%)) and clinical years (207 (47.2%)), with a smaller percentage in the internship category (19 (4.3%)). ORL involvement was reported in 159 (36.2%) of the participants.

Conclusion: The surgical specialty of ORL focuses mostly on conditions affecting the head, neck, nose, and ears. Since students found this specialty fascinating, we recommend that senior doctors make a greater effort to enlighten doctors-in-training about this field of expertise through lectures and campaigns at hospitals and universities.

## Introduction

Otorhinolaryngology (ORL) is a surgical subspecialty that focuses on diseases affecting the ears, nose, throat, head, and neck. ORL specialists treat patients from all age groups, ranging from neonates with airway challenges to elderly patients with head and neck cancer [1].

Knowledge of professional choices among medical students is important to ensure a balanced distribution of doctors in different specialties. Specialties lacking an adequate workforce need to be incentivized. In medical colleges, students encounter a broad range of specialties. Numerous factors are considered to guarantee a reasonable understanding of the burdens in each specialty, including personality characteristics, lifestyle, flexibility of work hours, and income [2-7].

Various factors influence the professional decisions of undergraduate students, such as individual passion for the specialty, future aspirations, career reputation, job movement, and salary expectations [8]. Published data suggest that medical students prefer certain specialties before graduation [9].

Studies have also shown that medical students frequently choose surgery, internal medicine, pediatrics, obstetrics, and gynecology [10]. Female doctors favored specialties where they could find flexibility in work hours, which gave them more time to spend with their families and friends. Male doctors exhibited an affinity for high-income specialties [10]. Furthermore, male doctors were more prone to studying and working abroad [11].

A study conducted among medical students in Najran, Saudi Arabia, found that no one chose ORL as the first choice [12]. Notably, short training in the ORL department leads to less exposure to the specialty, leading to less likelihood of fostering a preference for it [12]. Good teaching, exposure to a variety of diseases, and sufficient time spent in an ORL-head and neck surgery department are more likely to encourage choosing it as a future career [13].

The choice of a new specialty is constantly changing and affected by various factors. Therefore, it is necessary to understand what motivates medical students. This study aimed to explore factors affecting the choice of ORL-head and neck surgery as a future career among medical students at Umm Al-Qura University, Makkah, Saudi Arabia.

## Materials and methods

A cross-sectional study was performed from October to November 2023 via an online survey on social media to collect data on participants' demographics and the causes and factors affecting their preference for ORL-head and neck surgery as a future career. This study was conducted among medical students at Umm Al-Qura University, Makkah, Saudi Arabia, using validated questionnaires (English version).

The survey tool was prepared based on a review of existing literature. Using the Raosoft calculator, the sample size was determined to be more than 302 applicants, with a confidence interval of 95% and a level of significance (p-value) of 5%. In total, 439 participants completed the survey. We included male and female medical students in their second to sixth years and those undergoing internships.

The survey was composed of three parts. The first part included demographic details, including sex, age, and academic year. The second part of the questionnaire dealt with obtaining information about ear, nose, and throat (ENT) rotation, such as its duration and year, whether an objective structured clinical examination (OSCE) was part of it, and which doctor's operating room and the outpatient clinic the students spent the majority of their rotation time in. For students who considered ORL-head and neck surgery as one of their future specialty options, the third section of the questionnaire contained inquiries about the variables that influenced this choice.

This section of the survey was modified based on research conducted by Scott et al. [14]. Statistical analyses were performed using the RStudio software (R version 4.3.1). Categorical variables are expressed as frequencies and percentages. Multiple-response analysis was performed to analyze variables with multiple available responses. Factors associated with interest in ENT as a future specialty were explored using Pearson's chi-square test. Statistical significance was defined as p<0.05.

Ethical approval

The Institutional Research Board (IRB) (Biomedical Research Ethics Committee) at Umm Al-Qura University in Makkah City, Saudi Arabia, approved the study (IRB number: HAPO-02-K-012-2023-10-1812).

Before the study, all participants were provided with information regarding the study's objectives and were asked for their agreement. They were also told that their responses would be kept confidential.

## Results

Demographic and academic characteristics

In this study, we analyzed the responses of 439 medical students at Umm Al-Qura University, Makkah, Saudi Arabia. Females accounted for 339 (77.2%) of the participants.

As regards marital status, a majority of the participants reported being non-engaged (402 (91.6%)). Participants were evenly distributed across pre-clinical (213 (48.5%)) and clinical (207 (47.2%)) years, with a smaller percentage in the intern category (19 (4.3%)). Regarding academic performance, a substantial proportion of students had a GPA exceeding 3.70 out of 4 (291 (66.3%)). Notably, only 78 (17.8%) participants completed their ORL-head and neck rotations (Table 1).

**Table 1:** Demographic and academic characteristics N represents the number of participants and % the percentage of them. GPA: grade point average, ORL: otorhinolaryngology

Characteristic	N=439 (N (%))
Gender	
Male	100 (22.8%)
Female	339 (77.2%)
Marital status	
Non-engaged	402 (91.6%)
Engaged	37 (8.4%)
Academic year	
Pre-clinical	213 (48.5%)
Clinical	207 (47.2%)
Intern	19 (4.3%)
GPA (out of 4)	
<3.00	17 (3.9%)
3.00-3.39	37 (8.4%)
3.40-3.69	94 (21.4%)
>3.70	291 (66.3%)
Have you completed your ORL-head and neck rotation?	78 (17.8%)
Your preference regarding accompanying physician in ORL-head and neck surgery outpatient clinics and operation rooms	
Resident	144 (32.8%)
Specialist	136 (31.0%)
Consultant	159 (36.2%)

Interest in ORL and associated factors

**Figure 1:**
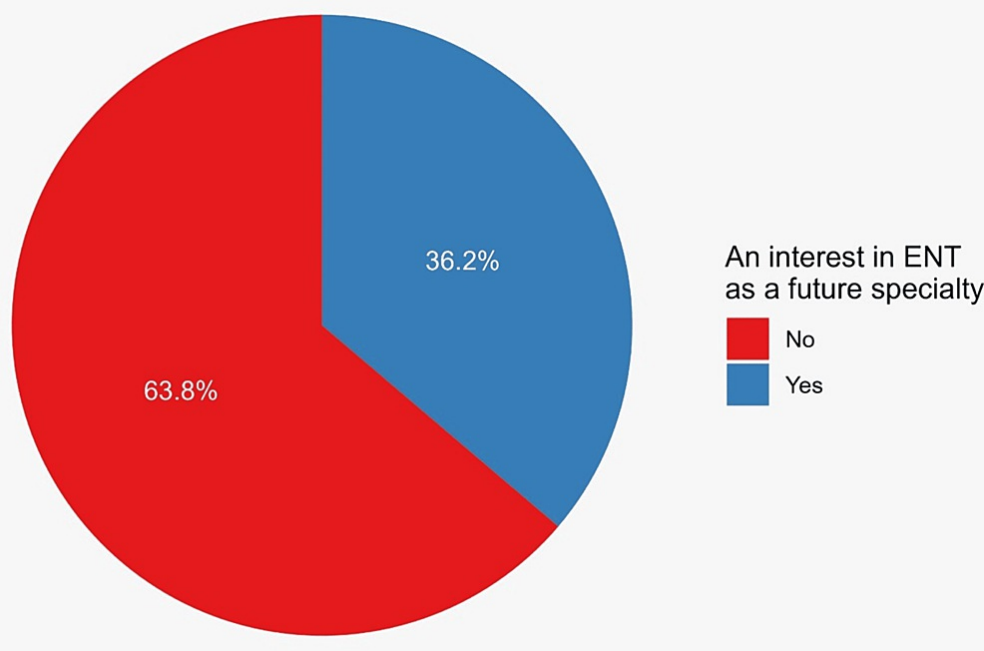
Proportions of participants with and without interests in ENT as a future specialty ENT: ear, nose, and throat

When asked about their future specialty preferences, 159 students expressed interest in ORL, representing 36.2% of the study sample (Figure 1).

In terms of accompanying physicians in ORL-head and neck surgery outpatient clinics and operating rooms, the highest preference was for consultants (61 (38.4%)). Interest in ORL was not significantly associated with any demographic or academic characteristic of the participants (Table 2).

**Table 2:** Factors associated with having an interest in ENT as a future specialty p-value is considered significant at p<0.05 and p<0.001. ENT: ear, nose, and throat, GPA: grade point average, ORL: otorhinolaryngology

Characteristic	Having an interest in ENT		p-value
	No (N=280) (N (%))	Yes (N=159) (N (%))	
Gender			0.773
Male	65 (23.2%)	35 (22.0%)	
Female	215 (76.8%)	124 (78.0%)	
Marital status			0.198
Non-engaged	260 (92.9%)	142 (89.3%)	
Engaged	20 (7.1%)	17 (10.7%)	
Academic year			0.113
Pre-clinical	128 (45.7%)	85 (53.5%)	
Clinical	142 (50.7%)	65 (40.9%)	
Intern	10 (3.6%)	9 (5.7%)	
GPA (out of 4)			0.294
<3.00	13 (4.6%)	4 (2.5%)	
3.00-3.39	26 (9.3%)	11 (6.9%)	
3.40-3.69	64 (22.9%)	30 (18.9%)	
>3.70	177 (63.2%)	114 (71.7%)	
Have you completed your ORL-head and neck rotation?	45 (16.1%)	33 (20.8%)	0.217
Your preference regarding accompanying physician in ORL-head and neck surgery outpatient clinics and operation rooms			0.776
Resident	94 (33.6%)	50 (31.4%)	
Specialist	88 (31.4%)	48 (30.2%)	
Consultant	98 (35.0%)	61 (38.4%)	

Perceptions regarding certain aspects of ORL

**Figure 2:**
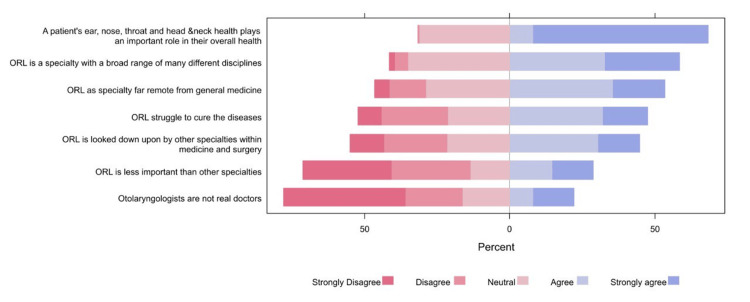
Participants' perceptions regarding certain aspects of ORL ORL: otorhinolaryngology

A majority of the participants agreed or strongly agreed with the statement that "a patient's ear, nose, throat, and head and neck health plays an important role in their overall health" (299 (68.3%)). While a considerable proportion acknowledged ORL as a specialty with a broad range of disciplines (256 (58.5%)), there was a notable concern about ORL being perceived as far removed from general medicine (234 (53.5%)), expressing agreement with the notion. Contrastingly, 92 (22.1%) and 126 (28.7%) students, respectively, agreed or strongly agreed with the perception that otolaryngologists were not real doctors and that ORL was less important than other specialties (Figure 2).

Perceived attractive and unattractive features in otolaryngologists' work

An analysis of the participants' perceptions of the most attractive features influencing their choice to work in ORL revealed that the majority strongly agreed or agreed with factors such as versatile and challenging work (283 (64.4%)), comprehensive doctor-patient relationships (286 (65.1%)), and the opportunity to meet people of different age groups and backgrounds (341 (77.7%)). Additionally, aspects such as rewarding work with grateful patients (345 (78.6%)) and the perception of a secure and respected job (339 (77.2%)) were highly endorsed. Conversely, when considering the factors that may deter participants from choosing ORL as a future specialty, the highest agreement was observed regarding long-term doctor-patient relationships with demanding patients (221 (50.4%)) and finding the work too challenging and difficult (216 (49.2%)) (Table 3).

**Table 3:** Description of the perceived attractive and unattractive features in otolaryngologists' work that could have made the participants choose to work or not work in ORL N represents the number of participants and % the percentage of them. ORL: otorhinolaryngology

Characteristic	Strongly disagree	Disagree	Neutral	Agree	Strongly agree
The most attractive features in otolaryngologists' work that could make you choose to work in ORL					
Long-term doctor-patient relationships	6 (1.4%)	56 (12.8%)	120 (27.3%)	138 (31.4%)	119 (27.1%)
Versatile, challenging work	2 (0.5%)	34 (7.7%)	120 (27.3%)	170 (38.7%)	113 (25.7%)
Comprehensive doctor-patient relationships	2 (0.5%)	29 (6.6%)	122 (27.8%)	170 (38.7%)	116 (26.4%)
A window into ordinary people's everyday life	0 (0.0%)	32 (7.3%)	117 (26.7%)	192 (43.7%)	98 (22.3%)
Opportunity to meet people of different age groups and various backgrounds	4 (0.9%)	19 (4.3%)	75 (17.1%)	201 (45.8%)	140 (31.9%)
Well-paid job	2 (0.5%)	11 (2.5%)	91 (20.7%)	160 (36.4%)	175 (39.9%)
Rewarding work, grateful patients	0 (0.0%)	6 (1.4%)	88 (20.0%)	164 (37.4%)	181 (41.2%)
Secure and respected job	0 (0.0%)	4 (0.9%)	96 (21.9%)	160 (36.4%)	179 (40.8%)
The most unattractive features in otolaryngologists' work that make you rethink the possibility of working as an otolaryngologist in the future					
Long-term doctor-patient relationships with demanding patients	19 (4.3%)	72 (16.4%)	127 (28.9%)	111 (25.3%)	110 (25.1%)
Work too challenging and difficult	21 (4.8%)	65 (14.8%)	137 (31.2%)	158 (36.0%)	58 (13.2%)
Work too routine and tedious	22 (5.0%)	58 (13.2%)	134 (30.5%)	148 (33.7%)	77 (17.5%)
Too much dealing with non-medical problems	29 (6.6%)	75 (17.1%)	132 (30.1%)	128 (29.2%)	75 (17.1%)
Too hasty and pressing work	17 (3.9%)	68 (15.5%)	167 (38.0%)	119 (27.1%)	68 (15.5%)
Poor-paid job	44 (10.0%)	89 (20.3%)	133 (30.3%)	82 (18.7%)	91 (20.7%)

Perceptions regarding the reasons for ORL preference and non-preference

Among students who did not prefer the ORL-head and neck specialty at Umm Al-Qura University, the primary cause cited was not finding the specialty interesting (250 (56.9%)). Other notable factors contributing to non-preference included a preference for other medical specialties (227 (51.7%)) and a lack of information (199 (45.3%)). However, for those preferring the ORL-head and neck specialty, the most frequently cited reason was acceptable hours of practice (214 (48.7%)). Additionally, flexibility in medicine (198 (45.1%)) and a stable/secure future (194 (44.2%)) were also significant factors influencing the preference for ORL (Table 4).

**Table 4:** Perceptions regarding the reasons for ORL preference and non-preference N represents the number of participants and % the percentage of them. ORL: otorhinolaryngology

Characteristic	N=439 (N (%))
Main cause of non-preference for ORL-head and neck specialty among students not-preferring ORL-head and neck specialty	
Bad surgical lifestyle	99 (22.6%)
Bad teaching	63 (14.4%)
Difficult surgery	68 (15.5%)
No information	199 (45.3%)
Prefer other surgical	143 (32.6%)
Require high grades	152 (34.6%)
Not interesting	250 (56.9%)
Prefer other medical specialty	227 (51.7%)
Main cause of preference for ORL-head and neck specialty among students preferring ORL-head and neck specialty	
Able to spend appropriate time with my family	172 (39.2%)
Acceptable on-call schedule	140 (31.9%)
Health promotion is important	50 (11.4%)
Experiences in health fields during medical school	63 (14.4%)
Focus on in-hospital care	72 (16.4%)
High income potential	152 (34.6%)
Keep options open	119 (27.1%)
Flexibility outside of medicine	107 (24.4%)
Research interest	111 (25.3%)
Focus on patients in community	69 (15.7%)
Good match to career	127 (28.9%)
Intervention results immediate	78 (17.8%)
Meaningful past experience	57 (13.0%)
Patient population is interesting	71 (16.2%)
Prefer medical to social problems	55 (12.5%)
Acceptable hours of practice	214 (48.7%)
Experiences with role models during medical school	66 (15.0%)
Stable/secure future	194 (44.2%)
Flexibility inside of medicine	198 (45.1%)

## Discussion

Choosing a postgraduate program is often considered one of the most challenging decisions for medical students. Several variables influence the decisions of medical students and interns regarding their future specialization.

This study aimed to examine the determinants of medical students' selection of ORL-head and neck surgery as a prospective specialty. A total of 439 medical students enrolled in the Faculty of Medicine, Umm Al-Qura University, Makkah, Saudi Arabia, participated in the survey.

Our study found that factors such as sex, marital status, academic year, GPA, and completion of the ORL-head and neck surgery rotation did not significantly affect students' preference for ORL-head and neck surgery as a future career. Similarly, another study showed that age, sex, and duration of rotation did not significantly affect students' preference for ORL-head and neck surgery [15]. Contrastingly, another study found that limited exposure prevented students from choosing ORL-head and neck surgery as a future specialty [16].

A study conducted in Nigeria found that none of the participants chose ORL-head and neck surgery as their preferred specialty [12]. Additionally, only 22 (6.6%) medical students in Saudi Arabia were interested in ORL-head and neck surgery [17]. Both studies considered this finding to be due to the limited exposure to the specialty in medical school. However, in this study, we found that 156 (36.2%) participants showed an interest in ORL-head and neck surgery as a future specialty, with a minority of the participants completing ORL-head and neck surgery rotation (78 (17.8%)).

Students' decisions to pursue surgical professions are affected by their early exposure to positive role models and professional and educational possibilities in surgery as a career field [7]. According to 78 (78%) medical students in a previous study, exposure to residents was the primary motivator for choosing ORL-head and neck surgery, highlighting the importance of resident-student relationships [13]. However, we found no impact of the physician's role on medical students' preference for accompanying them in ORL-head and neck surgery outpatient clinics or operating rooms. This finding may be due to differences in participants' experiences in outpatient clinics and operating rooms.

Regarding the perception of certain aspects of ORL-head and neck surgery, a large proportion of the medical students (29 (68.3%)) believed that ENT health was an important reflection of overall health. Additionally, a smaller proportion of respondents acknowledged that the ORL specialty encompasses a broad range of different disciplines, highlighting the importance of ORL in contributing to overall health; however, over half of the students (25 (58.5%)) agreed that ORL was far removed from general medicine. Additionally, 9-12 (22.1%-28.7%) agreed that doctors who chose ORL as their specialty were not real doctors. One contributing factor to this perception may be the low exposure to specialties throughout medical school. A survey conducted in the United Kingdom highlighted that 16% of the national selection candidates had not received any exposure to ENT during their medical curriculum or clinical foundation [18].

One of the most compelling aspects that motivated students to pursue ORL as a specialty was satisfaction with working with appreciative patients (345 (78.6%)). The broad spectrum of patients from diverse age groups, sexes, and backgrounds (341 (77.7%)) further contributes to the appeal of choosing ORL as a career. However, it is worth mentioning that the challenges associated with developing long-term doctor-patient relationships (221 (50.4%)), the level of difficulty, and the considerable effort required in the field (216 (49.2%)) are factors that students also consider when making decisions about their ORL career path.

The primary reason students at Umm Al-Qura University did not prefer ORL as a specialty included a lack of interest (250 (56.9%)). According to a study by Alamri et al. [19], this is one of the primary reasons students do not pursue ORL as a specialty. Other factors included a preference for other specialties (227 (51.7%)) and a lack of information regarding ORL (199 (45.3%)). For those preferring ORL, the main reasons were acceptable working hours (214 (48.7%)), flexibility in medicine (198 (45.1%)), and a stable future (194 (44.2%)).

Study strengths

This study offers valuable insights into the factors influencing medical students' decisions regarding ORL-head and neck surgeries. With a large sample size, these findings contribute to existing literature. The comprehensive analysis and practical implications provide actionable recommendations for promoting interest in this specialty.

Limitations

This study has some limitations, as it included a single university. Furthermore, we included all second-year undergraduates and medical interns with varying degrees of experience in ORL-head and neck surgery; also, a few interns participated in the study.

Recommendations

To promote interest in ORL-head and neck surgery as a specialty, enhancing exposure through early and comprehensive rotations, fostering positive role models, and integrating ORL-related topics into the medical curriculum are recommended. These measures can inspire students, shape their perceptions, and highlight the multidisciplinary nature of this field.

## Conclusions

This study sought to understand the factors that contribute to patients' decisions to specialize in ORL-head and neck surgery in Makkah, Saudi Arabia. A large portion of participants chose ORL for a variety of reasons, including its function in general health, its vast range of many different disciplines, and its perception as being far from general medicine, as indicated by 36.2% of the respondents. These findings suggest that senior physicians should work harder to educate juniors about their areas of expertise through campaigns and lectures at hospitals and universities.

## Appendices

Questionnaire

This survey is designed for research purposes only, and all data will be confidential and utilized solely for the research's aim. Also, it does not include any questions that may reveal your identity. No participant could be identified in person. Participation in the research is voluntary, and you have the right to participate in any of its stages (whenever you want) without losing any benefit you deserve. If you consent to participate in this survey, please be sure to answer all questions honestly. Conducting this research depends on your cooperation, which is highly appreciated. We thank you in advance for your time and participation in advancing science.

Table 5 and Table 6 show parts 1 and 2, respectively, of the questionnaire.

**Table 5:** Part 1 of the questionnaire GPA: grade point average

Characteristic
Q1: Gender?
Male
Female
Q2: Marital status?
Non-engaged
Engaged
Q3: Academic year?
Pre-clinical
Clinical
Intern
Q4: Your GPA (out of 4)?
<3.00
3.00-3.39
3.40-3.69
>3.70

**Table 6:** Part 2 of the questionnaire ORL: otorhinolaryngology, ENT: ear, nose, and throat

Q1: Have you completed your ORL-head and neck rotation?
o Yes
o No
Q2: Are you interested in ENT as a future specialty?
o Yes
o No
Q3: Your preference regarding accompanying a physician in ORL-head and neck surgery outpatient clinic and operation rooms?
o Resident
o Specialist
o Consultant

Question 4 is shown in Table 7.

**Table 7:** Q4 of part 2 of the questionnaire The above questions do not have a correct or incorrect answer but are intended to assess your opinion regarding certain aspects of ORL. ORL: otorhinolaryngology

Q4					
	Strongly agree	Agree	Natural	Disagree	Strongly disagree
A patient's ear, nose, throat, and head and neck health plays an important role in their overall health.					
ORL is a specialty far remote from general medicine.					
ORL is less important than other specialties.					
ORL is looked down upon by other specialties within medicine and surgery.					
ORL struggles to cure diseases.					
Otolaryngologists are not "real" doctors.					
ORL is a specialty with a broad range of many different disciplines.					

Question 5 is shown in Table 8.

**Table 8:** Q5 of part 2 of the questionnaire ORL: otorhinolaryngology

Q5: Which of the following would be the most attractive features in otolaryngologists' work that could make you choose to work in ORL?					
	Strongly agree	Agree	Natural	Disagree	Strongly disagree
Long-term doctor-patient relationships					
Versatile, challenging work					
Comprehensive doctor-patient relationships					
A window into ordinary people's everyday life					
Opportunity to meet people of different age groups and various backgrounds					
Well-paid job					
Rewarding work, grateful patients					
Secure and respected job					

Question 6 is shown in Table 9.

**Table 9:** Q6 of part 2 of the questionnaire

Q6: Which of the following would be the most unattractive features in otolaryngologists' work that make you rethink the possibility of working as an otolaryngologist in the future?					
	Strongly agree	Agree	Natural	Disagree	Strongly disagree
Long-term doctor-patient relationships with demanding patients					
Work too challenging and difficult					
Work too routine and tedious					
Too much dealing with non-medical problems					
Too hasty and pressing work					
Poor-paid job					
Work too lonely					

Table 10 presents part 3 of the questionnaire.

**Table 10:** Part 3 of the questionnaire ORL: otorhinolaryngology

Q1: What do you think is the main cause of non-preference for ORL-head and neck specialty among students not preferring ORL-head and neck specialty?
o Bad surgical lifestyle
o Not interesting
o Prefer other medical specialty
o No information
o Require high grades
o Prefer other surgical
o Difficult surgery
o Bad teaching
Q2: What do you think is the main cause of preference for ORL-head and neck specialty among students preferring ORL-head and neck specialty?
o Flexibility inside of medicine
o Acceptable hours of practice
o Stable/secure future
o Able to spend appropriate time with my family
o Good match to career
o Acceptable on-call schedule
o Keep options open
o High income potential
o Health promotion is important
o Intervention results immediate
o Experiences in health fields during medical school
o Flexibility outside of medicine
o Meaningful past experience
o Focus on patients in the community
o Focus on in-hospital care
o Patient population is interesting
o Research interest
o Experiences with role models during medical school
o Prefer medical to social problems
